# Transcriptomic Analysis of Pseudoscorpion Venom Reveals a Unique Cocktail Dominated by Enzymes and Protease Inhibitors

**DOI:** 10.3390/toxins10050207

**Published:** 2018-05-18

**Authors:** Carlos E. Santibáñez-López, Andrew Z. Ontano, Mark S. Harvey, Prashant P. Sharma

**Affiliations:** 1 Department of Integrative Biology, University of Wisconsin-Madison, 430 Lincoln Drive, Madison, WI 53706, USA; ontano@wisc.edu (A.Z.O.); prashant.sharma@wisc.edu (P.P.S.); 2 Posgrado en Ciencias Biológicas, Universidad Nacional Autónoma de México, Av. Universidad 3000, Coyoacán, Ciudad de México C.P. 04510, Mexico; 3 Department of Terrestrial Zoology, Western Australian Museum, Locked Bag 49, Welshpool DC, Western Australia 6986, Australia; Mark.Harvey@museum.wa.gov.au

**Keywords:** Arachnida, enzymes, kunitz-type inhibitors

## Abstract

Transcriptomic and genomic analyses have illuminated the diversity of venoms in three of the four venomous arachnid orders (scorpions, spiders, and ticks). To date, no venom gland transcriptome analysis has been available for pseudoscorpions, the fourth venomous arachnid lineage. To redress this gap, we sequenced an mRNA library generated from the venom glands of the species *Synsphyronus apimelus* (Garypidae). High-throughput sequencing by the Illumina protocol, followed by de novo assembly, resulted in a total of 238,331 transcripts. From those, we annotated 131 transcripts, which code for putative peptides/proteins with similar sequences to previously reported venom components available from different arachnid species in protein databases. Transcripts putatively coding for enzymes showed the richest diversity, followed by other venom components such as peptidase inhibitors, cysteine-rich peptides, and thyroglobulin 1-like peptides. Only 11 transcripts were found that code for putatively low molecular mass spider toxins. This study constitutes the first report of the diversity of components within pseudoscorpion venom.

## 1. Introduction

Pseudoscorpions, commonly known as false scorpions or book scorpions, are small arachnids (0.5 mm to 5 mm) that are similar to scorpions in that they bear a pair of chelate pedipalps (pincers), but lack the characteristic stinger-bearing metasoma (tail) [1]. These animals live in almost all terrestrial habitats, commonly in leaf litter or soil, but also in caves or littoral habitats [2]. Like many arachnid orders, pseudoscorpions appeared in the fossil record of the Devonian, with the oldest crown group fossils dating back to 390 Ma [3]. Their phylogenetic position remains controversial. Early studies (e.g., [4,5,6]) suggested pseudoscorpions were a sister group to either mites [5] or solifugids [6]. Comparatively recent phylogenomic analyses have revealed an array of unstable placements for this order: as a problematic long-branch taxon at the base of Arachnida, as the sister group to Arachnopulmonata (Scorpiones + Tetrapulmonata), or as the sister group to scorpions [7,8,9].

A large clade of pseudoscorpions (Iocheirata) possess one or two venom glands within the pedipalpal fingers (used to immobilize their prey); venom glands are missing in the less diverse superfamilies Feaelloidea and Chthonioidea [2,10]. They therefore represent one of the four venomous arachnid orders (together with Acari [ticks], Araneae [spiders], and Scorpiones [scorpions]). Surprisingly, and in contrast to the remaining venomous arachnid groups, the composition of pseudoscorpion venom remains unknown. Santos et al. [11] studied the effect of the crude venom from *Paratemnoides elongatus* on a rat cerebral cortex. Their findings were suggestive of the presence of selective compounds (e.g., neurotoxins) acting in l-glu and GABA dynamics, but no specific compounds were reported.

**Figure 1:**
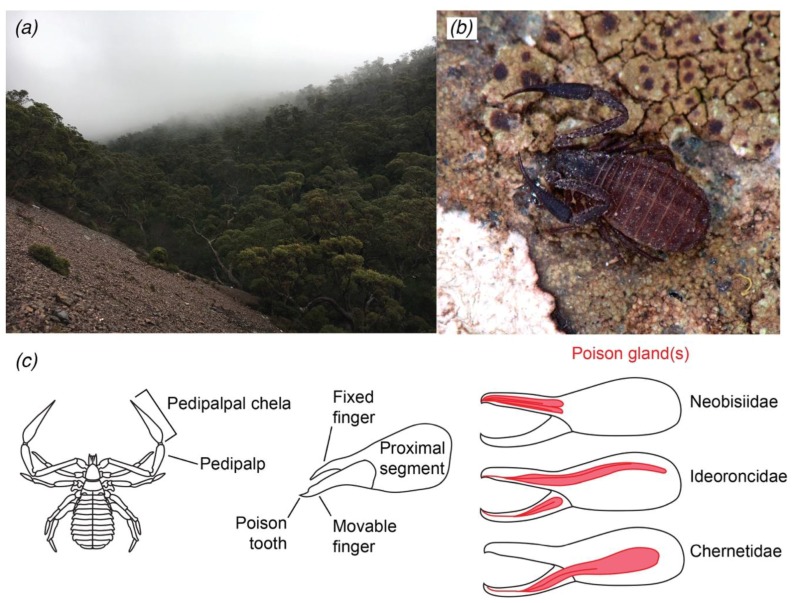
(**a**) Habitat of *Synsphyronus apimelus* in the Stirling Range National Park, Western Australia (photo by A.Z.O.). (**b**) Live habitus of adult *Synsphyronus apimelus* (photograph by G. Giribet, MCZ Database at https://mczbase.mcz.harvard.edu). (**c**) Schematic drawings showing the position of the venom glands in the pedipalpal chela of selected families of Iocheirata (the venomous pseudoscorpions), after References [22,23].

With the advent of high-throughput sequencing, studies on the diversity of peptidic components in scorpion and spider venom have become abundant. Through this approach, scorpion and spider venoms (see References [12,13]) have been discovered to contain many toxins that modulate the gating of ion channels, and also other components such as enzymes with phospholipase and hyaluronidase activities (e.g., [14,15,16,17,18]). Parallel inquiries via transcriptomic analysis of the salivary glands of ticks have revealed that these animals bear a great diversity of enzymes and protease inhibitors, but a low diversity of toxins [19,20,21]. As a first step toward discovering the diversity of venom components of Pseudoscorpiones, we present herein the first transcriptome analysis of the venom glands of the Western Australian species *Synsphyronus apimelus* (Garypidae; Figure 1). In addition, we selected transcripts coding for putative venom peptides and searched for orthologous sequences in two existing pseudoscorpion libraries (exemplars of the family Chernetidae) to assess evolutionary conservation of venom composition within the order.

## 2. Results

**Figure 2:**
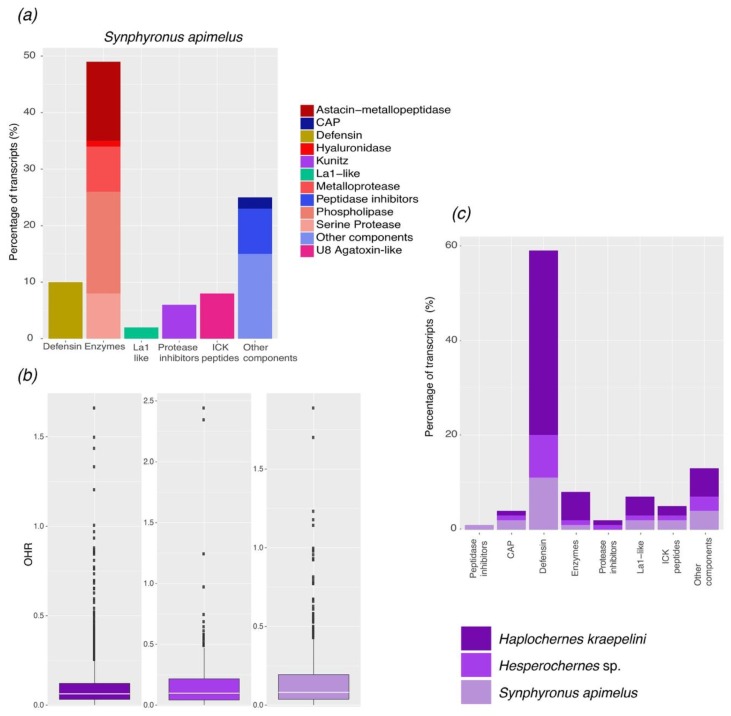
(**a**) Distribution of the annotated transcripts from the venom gland transcriptome of *S. apimelus* according to protein families and subfamilies. (**b**) Ortholog hit ratio (OHR) analysis showing the median (white line), and quartiles for three pseudoscorpion species. (**c**) Comparative distribution of the annotated transcripts from the transcriptomes of *S. apimelus*, *H. kraepelini,* and *Hesperochernes* sp.

The extraction of RNA from the pedipalpal chelae of *S. apimelus* yielded 3.367 µg of total RNA. After sequencing, assembly, and cleaning, 38,593,919 reads were obtained corresponding to 238,331 transcripts, 152,705 genes, and 53,483 peptides, with an N50 of 599 bp. From the transcripts, 37,148 were identified matching annotated genes listed in databases. Remarkably, only 54 were identified as matching arachnid sequences. This low number partly reflects the lack of annotated sequences in databases for arachnids, and especially so for pseudoscorpions [24]. In addition, 33,841 annotated genes were classified based on the Gene Ontology categories (GO-terms) [25,26]; the most abundant genes were those with molecular function (Figure S1). Finally, we detected 131 sequences (86 genes) which putatively code for venom components based on sequence similarity from UniProt, PFAM, or available literature (Figure 2a).

### 2.1. Transcriptomic Analysis

#### 2.1.1. ICK-Like Spider Venom Peptides

Toxins, generally the most widely studied venom fraction in all animals, are proteins classified according to their chemical class, biological origin, or target organ/ion channel [27]. Arachnid venoms are rich in toxins that modulate the opening of different ion channels in arthropods (mainly insects) and mammals. While high molecular mass toxins are more diverse in spider and tick venoms, low molecular mass toxins are far more diverse in scorpion venom. Here, we only found transcripts potentially coding for low molecular mass toxins in the pseudoscorpion. However, these were poorly represented in terms of sequence diversity, comprising only 11 transcripts (out of 131, 8%; Figure 2a). Within these transcripts, we discovered three sequences with 62–72% identity to the precursor of U8-agatoxin-like deduced from the genome of the spider *Parasteatoda tepidariorum*, seven sequences with 56–82% identity to the precursor of U8-agatoxin-like deduced from the genomic analysis of the scorpion *Centruroides sculpturatus*, and one sequence with 30% identity to the precursor of the U33-theraphotoxin-Cg1b deduced from cDNA cloned from the tarantula *Chilobrachys jingzhao*.

#### 2.1.2. Protease Inhibitors

Protease inhibitors, proteins capable of inhibiting the activity of proteolytic enzymes, may play an important role in the protection of toxins from unwanted degradation [28,29]. Kunitz-type inhibitors are frequently found in arthropod venoms. In the scorpion and spider venoms, these peptides have dual functions (see also Reference [30]) as protease inhibitors and potassium channel blockers (e.g., [31]). However, in ticks, mites, and insects, these peptides only act as serine protease inhibitors. In *S. apimelus*, we discovered eight sequences (6% of the total transcripts, Figure 2a) with different percentages of similarity (ranging from 46 to 64%) to five different precursors of Kunitz-type serine proteases reported from three spiders, one scorpion, and one insect.

#### 2.1.3. Enzymes

The most common enzymes in arachnid venom (i.e., mites, ticks, scorpions, and spiders) are hyaluronidases, metalloproteases, phospholipases, and serine proteases. Here, we report 62 sequences (48% of the total transcripts) coding putatively for the following enzymes: (a) one sequence with 34% identity to the precursor of a hyaluronidase deduced from cDNA cloned from the venom of the spider *Cupiennius salei*; (b) 17 sequences with different percentages of similarity (ranging from 42 to 73%) to six different precursors of Astacin-like metalloproteases reported from three spider, one scorpion, and one tick species; (c) seven sequences with identities ranging from 46 to 60% to two different precursors of Astacin-like metallopeptidases deduced from cDNA cloned from the venom gland of *Tityus serrulatus*; (d) two sequences of two different precursors of metalloproteinases reported from one spider and one insect; (e) 23 sequences with identities to four types of phospholipases (A2, D1, D2, and D3) reported from the venom of three spiders and two scorpions; and (f) 10 sequences with identities to six different precursors of serine proteases reported from the venom of one tick and two scorpions.

#### 2.1.4. Single Domain von Willebrand Factor Type C Peptides (La1-Like Peptides)

**Figure 3:**
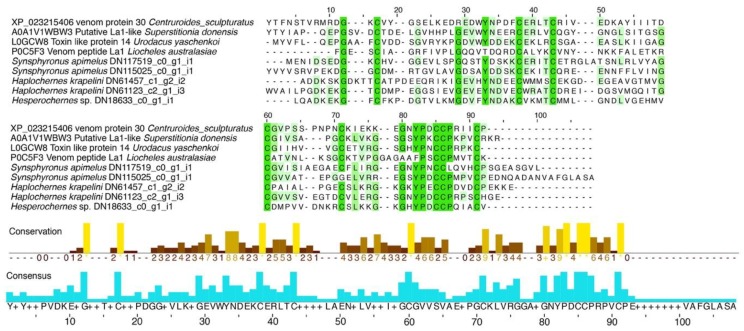
Multiple sequence alignment (MSA) of the peptide components with similarity to the single domain von Willebrand factor type C peptides (La1-like peptides) found in the transcriptome analysis of the venom gland of *S. apimelus*, *H. kraepelini,* and *Hesperochernes* sp. UniProt or GenBank numbers precede the peptide names. Percentage of identity between the MSA are highlighted in green. Below, histograms of the conservation and consensus of the MSA.

La1-like peptides have been found in scorpion venom, and recently in spider venom, but their function remains unclear [32,33,34]. In *S. apimelus*, we found two sequences with 40–46% identity to the toxin-like protein 14 isolated and deduced from cDNA cloned from the venom of the scorpion *Urodacus yaschenkoi* (Figure 3).

#### 2.1.5. Defensins

Defensins are peptides widely distributed throughout vertebrates, invertebrates, plants, and fungi, whose functions are determined by the displayed inter-cysteine loops or the residues in the core [35]. For example, arthropod defensins have antimicrobial activity [36]. However, recent studies have suggested that scorpion defensins share a common ancestor with scorpion ion channel toxins [37,38]. We discovered 13 sequences with identities corresponding to four different precursors of defensins reported from two ticks, one scorpion, and one spider.

#### 2.1.6. Other Components

Other transcripts potentially coding for venom proteins, including insulin-like growth factor binding protein, cysteine-rich secretory proteins, and peptidase inhibitors (not covered in the categories above) represent 26% of the transcripts annotated in this transcriptome analysis (Figure 2a). Among these, we found 15 with sequence similarity to the precursor of the U24 ctenitoxin Pn1 like that from *C. sculpturatus* and *P. tepidadorium*. Four sequences had similarity to a venom toxin peptide deduced from cDNA cloned from the venom of the scorpion *Hemiscorpius lepturus*. Additionally, we reported three sequences with less than 45% identity to a putative secreted salivary protein deduced from cDNA cloned from the tick *Ixodes scapularis*. Lastly, we found nine sequences with identities to two peptidase inhibitors reported from *C. sculpturatus* and *Stegodyphus mimosarum*.

### 2.2. Comparative Analysis of the Repertoire of Venom-Specific Transcripts in S. apimelus

The ortholog hit ratio (OHR) provides a proxy for the completeness of a transcriptome in terms of assembly coverage, with values above one suggesting insertions in the query sequence relative to the reference BLAST hit. Generally, most of the transcripts had a low OHR value (Figure 2b), suggesting that many of these transcripts contain relatively poorly conserved and/or unknown regions. Alternatively, the low OHR values could reflect low sequence coverage stemming from a large genome (to date, the size of a typical pseudoscorpion genome is unknown [24]). All venom categories reported here from the transcriptome of *S. apimelus* were found in the other two transcriptomes studied (Figure 2c). The number of genes coding for putatively venom proteins was slightly higher in the transcriptome of *Haplochernes kraepelini* (96 genes) but lower in the transcriptome of *Hesperochernes* sp. (73 genes). In all transcriptomes, enzymes were the most abundant proteins along with other venom components, such as the cysteine-rich secretory proteins and protease inhibitors (Figure 2c). Transcripts coding hyaluronidases were not found in the library of *Hesperochernes* sp. On the other hand, Kunitz-type inhibitors were poorly represented (5 to 10 genes). However, these transcripts correspond to several precursors of protease inhibitors reported from different arthropods (including insects and arachnids).

**Figure 4:**
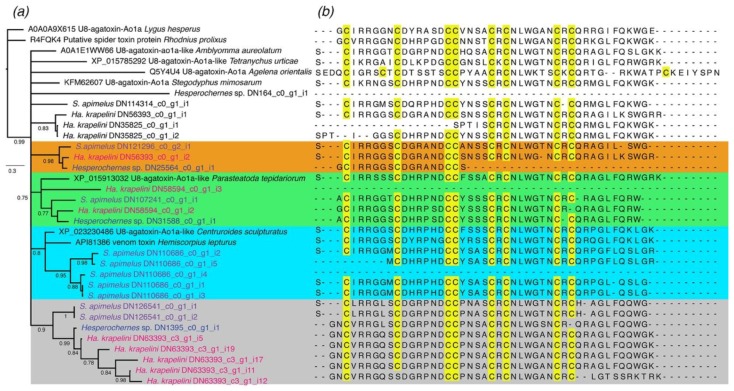
(**a**) Evolutionary tree of the U8-agatoxin-like peptides from a Bayesian analysis of 34 sequences reported from insects, and arachnids (including the sequences reported here). Posterior probabilities are indicated below nodes. (**b**) Multiple sequence alignment of the mature peptide predicted from the sequences used in the phylogenetic analysis. Those in blank had no mature peptide predicted and were represented only by the flanking region(s). Cysteine positions highlighted in yellow. Four orthogroup sequences from the three pseudoscorpion species are highlighted in colors (see text).

Low molecular mass spider toxins were poorly represented (in terms of diversity) in the three transcriptome libraries. Our phylogenetic analyses of U8-agatoxin-like peptides (ML and BI; Figure 4a) show the presence of three orthogroups, consisting of sequences from the three pseudoscorpion species. From these, one pseudoscorpion orthogroup was recovered with a U8-agatoxin-like homolog peptide, originally reported from the genomic analysis of *P. tepidariorum* with low nodal support (green clade in Figure 4b). The other two groups were pseudoscorpion-specific (orange and gray clades in Figure 4b). Finally, five transcripts from *S. apimelus* (representing one gene and five isoforms) clustered with the U8-agatoxin-like peptide from *C. sculpturatus* and another sequence reported from *Hemiscorpius lepturus*. No specific transcripts from any of the pseudoscorpion libraries clustered with peptides reported from tick venom (light blue clade in Figure 4b).

**Figure 5:**
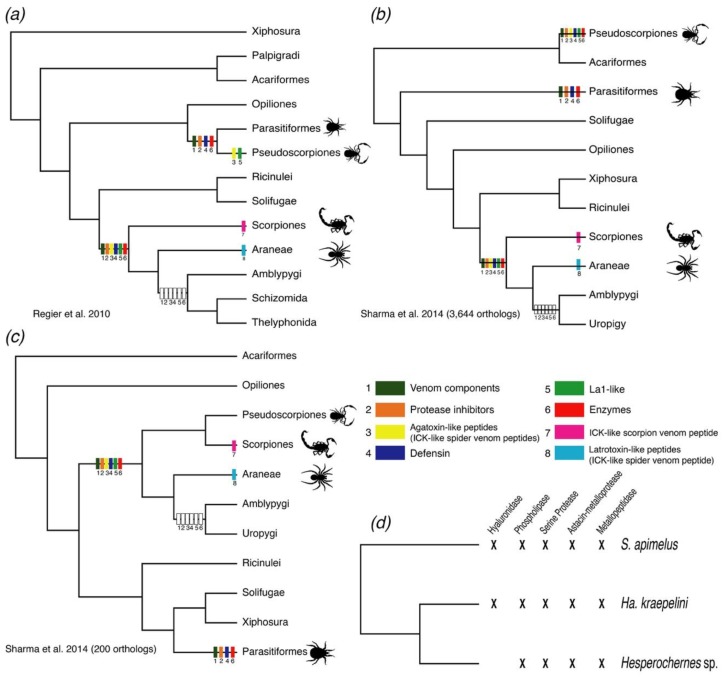
(**a**) Evolutionary hypotheses of the origins of venom components within Arachnida. (**a**–**c**) Tree topologies compiled from published sources [7,9] mapping each venom component by color. Blank squares indicate inferred secondary losses. (**d**) Phylogenetic relationships of the three pseudoscorpion species (compiled from References [1,2]) with the reported enzyme categories.

Our results suggest pseudoscorpion venom contains similar active peptides to those reported from spiders, ticks, and scorpions. To trace the evolutionary origin of the diversity of these components across arachnids, we mapped (using parsimony) eight categories of peptides with known function in the venom of spiders, scorpions, and ticks in the latest arachnid phylogeny [9] (Figure 5a–c). Enzymes (such as hyaluronidases and phospholipases), defensins, protease inhibitors, low molecular spider toxins (see below), and other venom components were shared by the four venomous arachnids. Within Pseudoscorpiones, all peptide categories were shared by the three libraries, except for the hyaluronidases (missing in the library of *Hesperochernes* sp., Figure 5d).

## 3. Discussion

Our high-quality pedipalpal transcriptome of *S. apimelus*, supported also by the analyses of two transcriptomes of other species, revealed for the first time the composition of pseudoscorpion venom. We found evidence for several components shared by the four venomous arachnid lineages, such as phospholipases, protease, and peptidase inhibitors. The presence of known peptidic toxins, such as those found in scorpion and spider venoms, were lowly represented. Our phylogenetic analyses of the low molecular mass spider toxins showed the presence of two different components unique to pseudoscorpions, and two components similar to the U8-agatoxin-like peptides from *P. tepidadorium* (spider) and from *C. sculpturatus* (scorpion). The function of the U8-agatoxin peptide (cloned from the spider *Agelena orientalis*) remains unknown [39]. However, its similarity to other agatoxins, a family of peptides including low mass molecular toxins with affinity to the sodium or calcium ion channels [40], suggest they might share similar functions. The only previous study on the effects of the crude venom of pseudoscorpion suggested the presence of putative neurotoxins of peptidic and nonpeptidic nature [11]. Whether the three groups found in pseudoscorpion venom are the culprit for neurotoxicity in rat brain is uncertain, because studies in spider venom have also shown the presence of polyamines targeting ionotropic glutamate receptors (e.g., [41,42]).

The composition of transcripts/genes in pseudoscorpion venom sheds light on the diversification of arachnid venom, both at the level of morphological sites of synthesis and molecular diversity. Spiders and ticks have their venom glands located anteriorly, injecting venom through the chelicerae (spiders) or salivary glands [19,43]. Scorpions on the other hand, possess venom glands located in the telson (the posterior-most part of the tail). Pseudoscorpions inject venom through the tips of the pedipalpal chela, with their venom glands located in the pedipalpal fingers or sometimes extending into the base of the chelal hand (Figure 1c). The evolution of venom glands within the four venomous arachnid orders is thus most likely the result of multiple independent evolutionary gains. The homology of the venom glands across the arachnids remains largely unexplored and may constitute an opportune target for cross-disciplinary studies of venom synthesis, evolution, and developmental genetics.

Resolving the phylogenetic position of Pseudoscorpiones would greatly refine the evolutionary context for arachnid venom. The origin of the venom peptide fraction has been suggested to be the recruitment of housekeeping genes into venom, followed by diversification and neofunctionalization (e.g., [44]). Following this reasoning, the phylogenetic position of Pseudoscorpiones is crucial to establishing the evolutionary relationship among venom components. Currently, the alternative phylogenetic positions of pseudoscorpions as (a) somehow related to the acarine orders (mites and ticks) or (b) more closely related to spiders and scorpions (Figure 5c), could be compatible with multiple scenarios. First, the most recent common ancestor (MRCA) of ticks and pseudoscorpions may have had these components, with separate gains at the base of Arachnopulmonata and secondary losses in non-venomous arachnopulmonate orders (Figure 5a). Alternatively, the similarities of pseudoscorpion and arachnopulmonate venom composition may be consistent with their closer phylogenetic relationship and a shared origin of venoms at the base of Pseudoscorpiones + Arachnopulmonata, a relationship supported in some phylogenomic analyses (Figure 5c). We also cannot rule out scenarios of multiple, independent gains of venom components in ticks, pseudoscorpions, and arachnopulmonates (Figure 5b). A more nuanced understanding of venom evolution within Arachnida is dependent upon resolving the phylogenetic position of Pseudoscorpiones and the constituent lineages of Acari, the mites and ticks.

## 4. Materials and Methods

Pseudoscorpion specimens were hand collected under stones in Stirling Range National Park, Western Australia (34°23′24″ S, 118°03′17″ E; 629 m elevation) on 18 August 2017 by A.Z.O., M.S.H., and P.P.S. (Figure 1). The pedipalpal chelae from 46 adult female and male specimens were dissected and transferred to 1.5 mL microcentrifuge tubes. Total RNA was extracted using the Trizol reagent (Ambion Life Technologies, Waltham, MA, USA). Library preparation and stranded mRNA sequencing followed protocols from the Biotechnology Center at the University of Wisconsin-Madison. Samples were run using an Illumina HiSeq2500 High Throughput platform with paired-end reads of 125 bp. Raw sequence reads can be found in the SRA database under the accession number SRR7062201 and the BioProject PRJNA453454. Adaptors were removed using Trimmomatic v. 0.36 [45] and the quality of cleaned raw reads was assessed with FastQC v. 0.11.5. [46]. Reads were assembled into contigs in a *de novo* fashion with Trinity v. 2.5 [47]. The quality of the assembly and basic statistics for the transcripts, genes, and isoforms were obtained using the *TrinityStats.pl* script. Assembled contigs were used as queries to search the UniProt database with the blastx and blastp algorithms; protein domains were identified with HMMER; and contigs were analyzed using Trinotate [47]. Additionally, to address the coverage of our transcripts, we calculated the ortholog hit ratio (OHR, [48,49,50]).

Selected transcripts with sequence similarity to venom components (e.g., from ticks, scorpions, spiders, or other arthropods) were used as queries to search UniProt and GenBank. From these new searches, matching sequences with lower expected (e) values, higher query cover values, or higher percentages of identity were selected as definitive matches. To contrast the venom components found in *S*. *apimelus* to other pseudoscorpion libraries (previously published), we assembled de novo the libraries of *Haplochernes kraepelini* and *Hesperochernes* sp. from raw reads downloaded from NBCI (accession numbers SRR1767661 and SRR1514877, respectively) following the same procedure as above. We calculated the OHR for their transcripts and used the selected venom transcripts from the library of *S. apimelus* as queries to search for orthologs in the other two libraries, using a phylogenetically informed orthology criterion, as implemented in UPhO [51]. The signal peptides and propeptides of these selected transcripts were determined with SpiderP from the Arachnoserver [52]. Multiple sequence alignments (MSA) of the relevant *S. apimelus* transcript-derived sequences with the corresponding input sets were obtained using MAFFT v. 7.0 [53]. Visualizations, conservations, and consensuses of MSA were obtain using Jalview v 2.10 [54].

To gain insights on the phylogenetic relationships of the transcripts with similarity to the low molecular mass spider toxins, we retrieved nine sequences which code, or putatively code, for U8 agatoxin and U8 agatoxin-like peptides from GenBank and UniProt. These sequences included two from cDNA cloned from two insect species; and seven deduced from cDNA cloned from seven arachnid species. Multiple sequence alignment for the full precursor was generated using MAFFT, resulting in a matrix consisting of 34 terminals and 208 amino acid sites. Maximum likelihood (ML) tree topologies were inferred in IQtree v 1.5.5 [55] using the PMB + Γ4 model, detected with ModelFinder [56] in IQtree, and by implementing 1000 ultrafast bootstrap resampling [57]. Bayesian inference (BI) analysis was performed with MrBayes 3.2.2 [58] using the JTT + Γ + I model, selected under the Bayesian information criterion using ProTest 3 [59]. Four runs, each with four Markov chains and a default distribution of chain temperatures, were implemented for 5 × 10^6^ generations. Convergence of each chain was assessed using Tracer v. 1.6 with 5 × 10^5^ generations discarded as burn-in.

## Supplementary Materials

The following are available online at http://www.mdpi.com/2072-6651/10/5/207/s1, Sequences reported from the library of *Synphyronus apimelus* are in fasta format.
