# Supplementary material for: Transcriptomic Analysis of Pseudoscorpion Venom Reveals a Unique Cocktail Dominated by Enzymes and Protease Inhibitors
Source: Toxins (Basel). 2018 May 18;10(5):207. doi: 10.3390/toxins10050207 (PMC5983263; doi:10.3390/toxins10050207)
Supplement: Supplementary file 1 [file toxins-10-00207-s001.zip › toxins-299884/Supplementary Figure.docx]

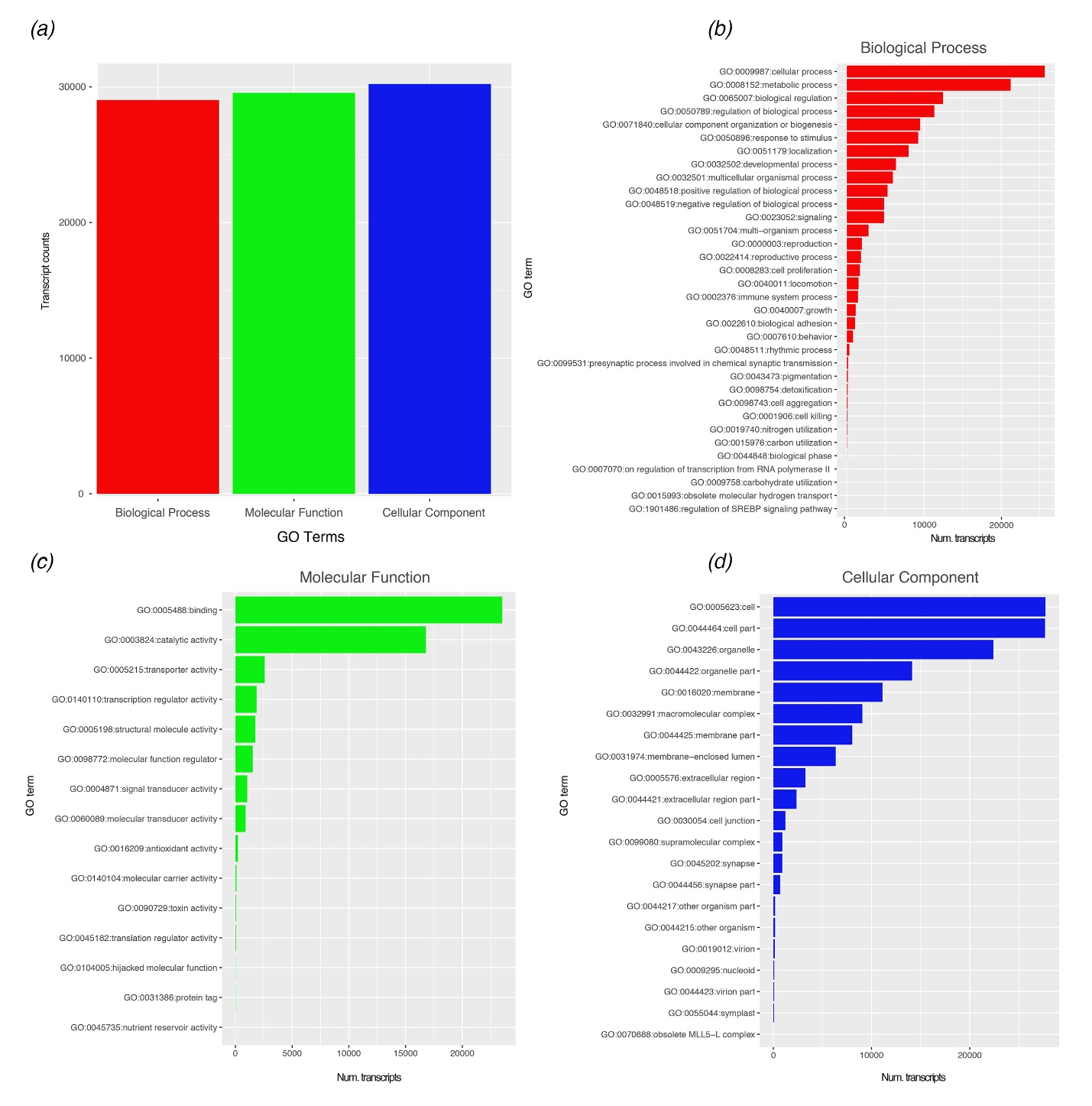


**Figure S1.** (a) Distribution of annotated transcripts from the venom gland transcriptome of S. apimelus according to Gene Ontology (GO) terms. (d-b) Distribution of the most represented categories within each GO term.
